# The 150 most important questions in cancer research and clinical oncology series: questions 31–39: Edited by *Chinese Journal of Cancer*

**DOI:** 10.1186/s40880-017-0215-6

**Published:** 2017-06-02

**Authors:** Chinese Journal of Cancer

**Affiliations:** Sun Yat-sen University Cancer Center, Guangzhou, 510060 Guangdong P. R. China

**Keywords:** Sarcoma, Intratumoral morphological heterogeneity, Pre-surgically differentiate irradiation-induced ulceration, Epidermal growth factor receptor, Non-small cell lung cancer, Epstein–Barr virus-related malignancy, Tumor heterogeneity, Prognostic classifier, Diffuse low-grade gliomas

## Abstract

To accelerate our endeavors to overcome cancer, *Chinese Journal of Cancer* has launched a program of publishing 150 most important questions in cancer research and clinical oncology. In this article, 9 more questions are presented as follows. Question 31: How does aging process inhibit the formation of sarcoma? Question 32: Is intratumoral morphological heterogeneity the consequence of tumor genomic instability or the cause of aggressive tumor behavior? Can we identify more aggressive tumors by computationally analyzing the morphological heterogeneity of the tumor tissues? Question 33: How to pre-surgically differentiate irradiation-induced ulceration from cancerous ulceration? Question 34: Why is epidermal growth factor receptor (EGFR) 19 Del-positive tumor more sensitive to targeted therapy than EGFR 21 L858R-positive tumor in patients with non-small cell lung cancer? Question 35: Can an Epstein–Barr virus vaccine be developed to reduce the incidence of EBV-related malignancies? Question 36: What is the unique feature in sarcoma vasculature that causes the intrinsic resistance of sarcoma against anti-angiogenic therapy? Question 37: How many ways can sarcoma cells protect themselves from the attacks of cytotoxic drugs? Question 38: How stable does the tumor heterogeneity remain along with cytotoxic chemotherapy? Question 39: How to generate a prognostic classifier for diffuse low-grade gliomas by integrating genetic and epigenetic signatures with histological features?

To accelerate our endeavors to overcome cancer, *Chinese Journal of Cancer* has launched a program of publishing 150 most important questions in cancer research and clinical oncology [1], with the first 30 questions been published in the last five issues [2–6]. In this article, Questions 31–39 are selected and presented. *Chinese Journal of Cancer* is still open to collect more key questions in cancer research and clinical oncology. Please send us your thoughtful questions to Ms. Ji Ruan via email: ruanji@sysucc.org.cn.

## Question 31: How does aging process inhibit the formation of sarcoma?

### Background and implications

It is known that a fully functioning immune system can wipe out transformed cells to prevent cancer formation. It is also widely accepted that young people usually have a better functioning immune system than the elderly. However, cervical sarcoma and osteosarcoma more commonly occur in young patients but rarely occur in the elderly. This phenomenon cannot be logically explained by the theory of escaping immunosurveillance of the cancer cells. A more plausible hypothesis is that the aging process of human being could somehow inhibit or prevent the initiation of sarcoma. Unveiling the underlying aging-triggered inhibitory mechanisms against sarcoma formation would open a window into better prevention and treatment of these fatal diseases.

#### Submitter

Wei-Jie Tian.

#### Affiliation and email

Department of Obstetrics and Gynecology, Guizhou Provincial People’s Hospital, Guiyang, Guizhou, P. R. China.

ajie666@foxmail.com

## Question 32

Question 1: Is intratumoral morphological heterogeneity the consequence of tumor genomic instability or the cause of aggressive tumor behavior?

Question 2: Can we identify more aggressive tumors by computationally analyzing the morphological heterogeneity of the tumor tissues?

### Background and implications

Intratumoral morphological heterogeneity has been observed in almost all types of solid tumor. From the perspective of differentiation, the better differentiated tumors are less heterogeneous in morphology and usually associated with less aggressive tumor behavior in terms of invading the surrounding normal tissue and spreading to distant organs, whereas the undifferentiated tumors usually harbor more obvious morphological heterogeneity and often posses more malignant behavior. However, it is undetermined how the morphological heterogeneity contributes to the aggressive tumor behavior and whether it is just simply the consequence of tumor genomic instability.

Careful observation of primary tumor nests (e.g., clear cell renal cell carcinoma) reveals that the tumor cells are usually smaller and expressing CD44 (a stem cell marker) in a higher level in the invasion margin than in the center of a nest. We are wondering whether we can determine the aggressive ability of the tumors by quantifying the morphological heterogeneity of the tumor through computational image analyses.

Answering the above two questions will dramatically improve the quality of pathologic practice and prognostic prediction in the era of precision medicine.

#### Submitter

Jing-Ping Yun.

#### Affiliation and email

Department of Pathology, Sun Yat-sen University Cancer Center, Guangzhou, Guangdong, P. R. China.

yunjp@sysucc.org.cn

## Question 33: How to pre-surgically differentiate irradiation-induced ulceration from cancerous ulceration?

### Background and implications

Postradiation nasopharyngeal necrosis often causes severe headache, foul odor, and epistaxis, which severely affects the quality of life of the patients. Both irradiation and cancer relapse could cause postradiation nasopharyngeal necrosis, and the treatment methods are completely different. Therefore, differentiating irradiation-induced ulceration from cancerous ulceration is extremely critical before choosing the treatment method. The pathologic result is the gold standard to make the diagnosis of postradiation nasopharyngeal necrosis, but we find a new phenomenon that superficial histopathologic examination does not provide precise results. Superficial histopathologic examination shows negative result, but the tissue from the bottom of the necrosis shows positive result in a few patients. We realize that only the tissue from the bottom of the necrosis can be useful for final diagnosis, but this tissue can only be obtained after surgical intervention. In current clinical practice, there is no satisfying examination method to efficiently identify the nature of irradiation-induced ulceration or cancerous ulceration except surgical intervention, even though using positron emission tomography-computed tomography. Developing a reliable method to differentiate irradiation-induced ulceration from cancerous ulceration would allow us to make a right choice of treating postradiation nasopharyngeal necrosis.

#### Submitter

Ming-Yuan Chen.

#### Affiliation and email

Sun Yat-sen University Cancer Center, Guangzhou, Guangdong, P. R. China.

chenmy@sysucc.org.cn

## Question 34: Why is epidermal growth factor receptor (EGFR) 19 Del-positive tumor more sensitive to targeted therapy than EGFR 21 L858R-positive tumor in patients with non-small cell lung cancer (NSCLC)?

### Background and implications

Among the patients with advanced NSCLC who underwent EGFR-tyrosine kinase inhibitor (EGFR-TKI) treatment, the patients with EGFR exon 19 deletion (19 Del)-positive tumor had a higher objective response rate, longer progression-free survival duration, and longer overall survival duration than those with exon 21 L858R mutation (21 L858R)-positive tumor. However, how EGFR 19 Del contributes to better treatment response remains undetermined. Moreover, whether these two types of genetic alterations can shift to each other is not clear. Determining the genomic backgrounds and biological roles of EGFR 19 Del and 21 L858R in NSCLC will be very helpful for personalized targeted therapy for EGFR-positive NSCLC.

#### Submitters

Yaxiong Zhang and Li Zhang.

#### Affiliation and email

Sun Yat-sen University Cancer Center, Guangzhou, Guangdong, P. R. China.

zhangyx@sysucc.org.cn; zhangli@sysucc.org.cn

## Question 35: Can an Epstein–Barr virus (EBV) vaccine be developed to reduce the incidence of EBV-related malignancies?

### Background and implications

EBV is one of the most common viruses and infects more than 95% adults worldwide. EBV infection causes infectious mononucleosis. As an oncogenic virus, EBV infection is associated with lymphoid malignancies, such as Hodgkin lymphoma, Burkitt lymphoma, and lymphoma in transplant recipients, and epithelial cell cancers, such as gastric carcinoma and nasopharyngeal carcinoma. It is believed that EBV infection contributes to nearly 200,000 new cases of cancers each year worldwide. Moreover, the number of serum EBV DNA copies is an effective prognostic indicator for nasopharyngeal carcinoma patients in high incidence areas. An EBV vaccine might prevent infection and disease or might reduce the viral load after primary infection to reduce the incidence of infectious mononucleosis or malignancies associated with EBV infection.

#### Submitters

Wei Bu and Jeffrey I. Cohen.

#### Affiliation and email

Laboratory of Infectious Diseases, National Institute of Allergy and Infectious Diseases, Bethesda, Maryland, USA.

buw@niaid.nih.gov; jcohen@niaid.nih.gov

## Question 36: What is the unique feature in sarcoma vasculature that causes the intrinsic resistance of sarcoma against anti-angiogenic therapy?

### Background and implications

Carcinomas commonly metastasize via the lymphatic system followed by the involvement of the blood vessel system, whereas sarcomas commonly spread via the blood vessel system in the first place. Current anti-angiogenic drugs (e.g., vascular endothelial growth factor signaling inhibitors) are believed to be able to inhibit tumor angiogenesis in some extents, and they have shown modest effects on carcinoma. However, most anti-angiogenic drugs are not effective in sarcoma as expected. The heterogeneity of tumor vasculature has been partially revealed. Sarcoma vasculature seems much more complicated than the vasculature of carcinoma. It is therefore worthy of further explorations to elucidate the unique feature in sarcoma vasculature responsible for the intrinsic resistance against anti-angiogenic therapy.

#### Submitter

Jilong Yang.

#### Affiliation and email

Tianjin Medical University Cancer Hospital & Institute, Tianjin, P. R. China.

yangjilong@tjmuch.com

## Question 37: How many ways can sarcoma cells protect themselves from the attacks of cytotoxic drugs?

### Background and implications

Sarcoma is a rare tumor type mainly metastasizing to the lung. Chemotherapy plays an important role in the treatment of advanced-stage sarcomas. However, the effect of current chemotherapeutic drugs on sarcoma is very poor, and chemotherapy resistance frequently occurs in almost all of the patients with sarcoma. The underlying mechanisms of sarcoma resistance against cytotoxic drugs are poorly known. To elucidate the mechanisms underlying sarcoma resistance against cytotoxic drugs will be very helpful for future drug development aiming to enhance the effect of anti-sarcoma chemotherapy.

#### Submitter

Jilong Yang.

#### Affiliation and email

Tianjin Medical University Cancer Hospital & Institute, Tianjin, P. R. China.

yangjilong@tjmuch.com

## Question 38: How stable does the tumor heterogeneity remain along with cytotoxic chemotherapy?

### Background and implications

Massive evidence has indicated that there are intertumourous and intratumourous heterogeneities in a variety of types of primary tumor. Tumor heterogeneity has not only been indentified in histological level but also been recognized in genetic and epigenetic levels. Importantly, tumor heterogeneity is considered a crucial mechanism underlying chemotherapy resistance. A wealth of studies suggested that the heterogeneity of targeted molecules, including V-Ki-ras2 Kirsten rat sarcoma viral oncogene homolog (KRAS), serine/threonine-protein kinase B-Raf (BRAF), and epidermal growth factor receptor (EGFR), promotes chemotherapy resistance of the tumor. However, increasing evidence has also shown that cytotoxic chemotherapy has the potential to induce genomic instability, which could result in more heterogeneity. Therefore, it is critical to know how stable the heterogeneity of a particular tumor remains upon multiple cycles of different chemotherapy regimens, which would facilitate our understanding on the occurrence of inevitable treatment resistance in most cancer patients, as well as better selection of more effective chemotherapy regimens for these patients.

#### Submitter

Chunlin Ou.

#### Affiliation and email

Central South University Cancer Research Institute, Changsha, Hunan, P. R. China.

ouchunlin@csu.edu.cn

## Question 39: How to generate a prognostic classifier for diffuse low-grade gliomas (LGGs) by integrating genetic and epigenetic signatures with histological features?

### Background and implications

Diffuse LGGs include World Health Organization (WHO) grade II astrocytoma, oligodendroglioma, and oligoastrocytoma, which usually exhibit relatively slow growth and are infiltrative brain tumors. The neoplastic cells of LGGs diffusely infiltrate to the surrounding/adjacent brain parenchyma (up to 3–4 cm from the “margin” of tumor resection), making it impossible to complete the surgical resection or neoplasia excision. Diagnosis is usually performed based on histological analysis; however, histological/phenotypic features often fail to offer sharp stratification between different grades of infiltrative tumors with astrocytic or oligodendroglial origin. In this context, there is increasing emphasis that the distinctive genetic signatures, especially those identified by comprehensive integrative analysis by The Cancer Genome Atlas (TCGA) platforms [1–4], and defined genetic entities suggested by the 2016 WHO grading system [5], should be used for circumscribed diagnosis.

Diffuse LGGs are characterized by mutually exclusive telomerase reverse transcriptase (*TERT*) and *ATRX* mutations, one of the best defined characteristic gene mutations such as isocitrate dehydrogenase 1,2 (*IDH1/2*) and tumor protein p53 (*TP53*) mutations and the combined deletion of 1p/19q regions. The *IDH* mutations are very early genetic events and are frequent in diffuse gliomas. Additionally, LGGs exhibit distinctly different CpG island methylator phenotype (G-CIMP). Of particular note, the G-CIMP status is strongly associated with *IDH1* somatic mutations and is more prevalent in diffuse gliomas [6, 7]. Followed by the mutations in characteristic genes, the acquisition of epigenetic modifications may reinforce the classification criteria of LGG subtypes with astrocytic and oligodendroglial origins which otherwise are less likely to be established by histological features alone.

It is therefore clinically important to generate a prognostic classifier to better predict the sensitivity of LGG to radiotherapy and chemotherapy and therefore predict the prognosis of LGG patients. This standardized and rigorously validated classifier by integrating genetic, epigenetic, and histological features of LGG should be superior to histological classification alone.

**References**

1. Eckel-Passow JE, et al. Glioma groups based on 1p/19q, IDH, and TERT promoter mutations in tumors. N Engl J Med. 2015;372(26):2499–508.
2. Cancer Genome Atlas Research Network. Comprehensive, integrative genomic analysis of diffuse lower-grade gliomas. N Engl J Med. 2015;372(26):2481–98.
3. Ceccarelli M, et al. Molecular profiling reveals biologically discrete subsets and pathways of progression in diffuse glioma. Cell. 2016;164(3):550–63.
4. Suzuki H, et al. Mutational landscape and clonal architecture in grade II and III gliomas. Nat Genet. 2015;47(5):458–68.
5. Louis DN, et al. The 2016 World Health Organization classification of tumors of the central nervous system: a summary. Acta Neuropathol. 2016;131(6):803–20.
6. Huse JT, Aldape KD. The evolving role of molecular markers in the diagnosis and management of diffuse glioma. Clin Cancer Res. 2014;20(22):5601–11.
7. Noushmehr H, et al. Identification of a CpG island methylator phenotype that defines a distinct subgroup of glioma. Cancer Cell. 2010;17(5):510–22.

#### Submitters

Farah Fatima and Muhammad Nawaz.

#### Affiliation and email

Ribeirão Preto Medical School, Av. Bandeirantes 3900, Ribeirão Preto 14049-900, University of São Paulo, Brazil.

farah.fatima00@gmail.com; nawazm.edu@gmail.com
